# Outpatient Management of Imported Nonsevere Falciparum Malaria in Adults in the United Kingdom: A Multisite Evaluation of Clinical Safety and Costing Analysis

**DOI:** 10.1093/ofid/ofag443

**Published:** 2026-07-22

**Authors:** James Milburn, Anne Williamson, Sneha Baljekar, Raissa Rachman, Anna Daunt, Naomi A Richardson, Flora Olcott, Clive Rodrigues, Rajeni Thangarajah, Shunmay Yeung, Mauricio Arias, Clara Jennings, Ashwin Delmonte Sen, John L Klein, Anand Odedra, Nathan Jacobs, Cecilia Drapeau, Clare van Halsema, Geraldine O’Hara, Anna Checkley

**Affiliations:** Department of Infectious Diseases, University College London Hospitals NHS Foundation Trust, London, UK; Department of Infection, Guy's and St Thomas’ Hospital NHS Foundation Trust, London, UK; Queen Mary and Barts Health Tuberculosis Centre, Blizard Institute, London, UK; Department of Infectious Diseases, University College London Hospitals NHS Foundation Trust, London, UK; Department of Infection, Guy's and St Thomas’ Hospital NHS Foundation Trust, London, UK; Department of Infection, Guy's and St Thomas’ Hospital NHS Foundation Trust, London, UK; Regional Infectious Diseases Unit, Manchester University NHS Foundation Trust, Manchester, UK; North Middlesex University Hospital, London, UK; Department of Infectious Sciences, King's College Hospital NHS Foundation Trust, London, UK; Department of Infection, Guy's and St Thomas’ Hospital NHS Foundation Trust, London, UK; Department of Pharmacy, Guy's & St Thomas’ NHS Foundation Trust, London, UK; Department of Global Health and Development, London School of Hygiene and Tropical Medicine, London, UK; Department of Clinical Research, London School of Hygiene and Tropical Medicine, London, UK; Department of Infectious Sciences, King's College Hospital NHS Foundation Trust, London, UK; Department of Infectious Sciences, King's College Hospital NHS Foundation Trust, London, UK; Department of Infectious Diseases, University College London Hospitals NHS Foundation Trust, London, UK; Department of Infection, Guy's and St Thomas’ Hospital NHS Foundation Trust, London, UK; Department of Infectious Diseases, University College London Hospitals NHS Foundation Trust, London, UK; Regional Infectious Diseases Unit, Manchester University NHS Foundation Trust, Manchester, UK; North Middlesex University Hospital, London, UK; Regional Infectious Diseases Unit, Manchester University NHS Foundation Trust, Manchester, UK; Department of Infection, Guy's and St Thomas’ Hospital NHS Foundation Trust, London, UK; Department of Infectious Diseases, University College London Hospitals NHS Foundation Trust, London, UK; Department of Clinical Research, London School of Hygiene and Tropical Medicine, London, UK

**Keywords:** ambulatory, artemisinin combination therapy, malaria, outpatient treatment

## Abstract

**Background:**

Imported falciparum malaria represents a significant health care burden disproportionate to its incidence. UK guidelines have traditionally recommended admission for all cases of nonsevere falciparum malaria. However, it is recognized that cases of nonsevere malaria meeting appropriate clinical criteria do not require additional supportive treatment and can be managed safely outpatient. Routine inpatient management may increase reluctance to present to hospital early and increase health care system and patient costs.

**Methods:**

We conducted a prospective multisite cohort study across 5 UK centers from 2019 to 2024, evaluating outpatient management of adults with nonsevere falciparum malaria. One hundred twenty-one patients received outpatient treatment with artemether-lumefantrine (AL) for 3 days, with structured follow-up including clinical review and/or repeat malaria testing. A micro-costed subanalysis of 2 sites compared the cost of care of outpatient treatment with a control group, defined as nonsevere malaria cases who were clinically similar but admitted overnight.

**Results:**

Clinical or parasitological cure within 2 weeks of initial blood film was achieved in 91% (99/109) of patients receiving outpatient artemether-lumefantrine therapy with appropriate follow-up. No escalations to high-dependency or intensive care and no deaths occurred during the study period. Among those requiring further intervention, 50% (5/10) did not complete the initial course. A cost analysis of outpatients vs patients admitted for nonclinical reasons demonstrated significant savings, with median costs of £1248 and £1792, respectively.

**Conclusions:**

Outpatient management of carefully selected patients with nonsevere falciparum malaria appears clinically safe and economically justified. These findings support development of standardized outpatient protocols for nonsevere falciparum malaria, potentially reducing health care costs while maintaining patient safety.

Malaria caused by *Plasmodium falciparum* remains one of the most common and potentially fatal imported infections and should be considered in all febrile patients returning from malaria-endemic countries. Cases of malaria in the United Kingdom have risen above prepandemic levels, with 2106 cases in 2023, and the vast majority of these are caused by *P. falciparum* [1]. The rapid diagnosis of malaria and recognition of clinical severity are crucial to delivery of prompt, effective therapy to prevent morbidity and mortality [2, 3]. Severe malaria is a medical emergency; however, it is recognized that there is a subset of patients with nonsevere falciparum malaria who are clinically stable on presentation, do not require additional supportive treatment, and respond well to oral therapy alone [4–7]. These patients have traditionally been admitted for treatment in the United Kingdom due to a lack of robust evidence demonstrating that outpatient management of nonsevere malaria in nonendemic countries is safe and clinically effective [8].

In malaria-endemic countries, outpatient management of nonsevere falciparum malaria is well established and recommended in World Health Organization (WHO) guidelines [9–11]. Data on the safety of outpatient management in nonendemic countries are limited, making the development of guidelines for nonimmune populations more challenging. In endemic regions, individuals can acquire partial immunity through repeated exposure to the malaria parasite, which reduces the severity of subsequent infections; patients living in nonendemic regions lack this immunity, contributing to differences in disease progression and management considerations [12, 13]. The few studies on outpatient treatment of nonsevere malaria in nonendemic countries have significant variability in their inclusion criteria and use antimalarial treatment that has now been superseded by more effective therapies [5–7, 14]. This is reflected in variability in clinical practice and in guidelines [12]. National guidelines in the United Kingdom from 2016 advocate for admission of all cases of falciparum malaria regardless of clinical assessment of severity [8]. However, many UK hospitals now routinely manage selected cases of nonsevere falciparum malaria outpatient [5, 12].

Safe and effective oral therapies are available for the treatment of nonsevere falciparum malaria. In many endemic regions, individuals are accustomed to seeking care at pharmacies, community health centers, or through self-treatment with readily available antimalarials, rather than hospital admission [15].

When individuals who have previously lived in endemic areas reside in a nonendemic area, a reluctance to present to hospital early with malaria symptoms may result from a perception that inpatient treatment may be mandated [16]. Safely aligning UK treatment pathways with familiar practices from endemic settings including outpatient management may encourage patients to seek care earlier, reducing delays. By avoiding unnecessary admissions, outpatient management may reduce the financial burden on health systems and patients, reduce exposure to health care–associated infections, and alleviate pressure on already overstretched health care services. However, data on the safety of outpatient management of nonsevere falciparum malaria in nonendemic settings remain scarce, and comprehensive health economic analyses evaluating its impact are lacking.

We present the first multisite study assessing the safety of outpatient management of nonsevere falciparum malaria with oral artemisinin combination therapy in a non-malaria-endemic country with accompanying health economic analysis comparing inpatient vs outpatient management.

## METHODS

### Study Design

This was a prospective multisite study assessing the safety of outpatient management of nonsevere falciparum malaria. Data from the Malaria Reference Laboratory (MRL) were used to identify the 10 National Health Service (NHS) trusts in the United Kingdom with the highest number of *P. falciparum* malaria notifications in 2017. All were invited to participate, and 5 agreed to take part. The participating sites were Guy's and St. Thomas’ NHS Foundation Trust (GSTT), the Hospital for Tropical Diseases (HTD), King's College Hospital NHS Foundation Trust (KCH), North Manchester General Hospital (NMGH), and North Middlesex University Hospital (NMUH).

Consensus guidelines for outpatient management of nonsevere falciparum malaria were established for 3 sites without outpatient treatment pathways in place. Guidelines were based on existing guidelines from international settings and expert opinion. These guidelines defined the inclusion and exclusion criteria for the study (Table 1). Two sites already had established outpatient pathways, and these were reviewed to ensure they aligned with the consensus guidelines (Supplementary Table 1).

**Table 1.** Inclusion and Exclusion Criteria for Outpatient Management of Nonsevere Falciparum Malaria

Inclusion Criteria	
Infection with *Plasmodium falciparum* confirmed on blood film	
Treated with oral antimalarials only with no prior intravenous antimalarials	
Exclusion Criteria	
Age	<16 or >60
Pregnancy	Pregnant people excluded
Immunity	Not born in malaria-endemic area within Sub-Saharan Africa
Comorbidities	Immunosuppression/poorly controlled diabetes mellitus/chronic kidney disease/chronic liver disease/splenectomy/other significant comorbidity
Laboratory parameters	Hemoglobin <100 g/dL
	Platelets <40 platelets/μL
	Creatinine >150 μmol/L
	Bilirubin >50 μmol/L
	Glucose <3.9 or >15
Parasitology results	>2% *P. falciparum* parasitemia or schizonts or parasitemia unavailable
Vomiting/intolerance	Does not tolerate first dose of oral antimalarial or is unable to take medication orally
Social circumstances	No responsible adult at home
Other	Travel to area with high rates of artemisinin resistance

The primary outcome was evidence of clinical or parasitological cure, defined as either complete resolution of symptoms on clinical review or a negative malaria blood film within 2 weeks of the initial blood film. Secondary outcomes were unscheduled health care visits due to malaria and repeat treatment or extensions of treatment for falciparum malaria.

### Patient Enrollment and Data Collection

Data were collected prospectively from consecutive adult patients (age ≥16 years) between December 2019 and December 2023 at 4 sites. At 1 site, the necessary data were recorded in electronic health records as part of routine clinical practice. Subsequently, the site joined the study, and data were retrospectively collected from consecutive patients presenting between 2022 and 2024. At 2 sites (GSTT and NMUH), outpatient treatment pathways were established before this study, but data collection began on the dates specified. Enrollment dates varied across study sites. Pseudo-anonymized clinical, ethnic, laboratory, and outcome data were captured from patient records using standardized data capture forms. The timing of follow-up varied between trusts; however, all scheduled follow-up within a 2-week window was captured. Anonymized data from all sites were centralized at HTD and stored on a secure server. Data sharing agreements between all study sites and HTD were in place, and each trust undertook a Data Protection Impact Assessment (details available on request). Descriptive analyses using Stata 18 were used to describe patient demographics, travel history, clinical characteristics, treatment adherence, and outcomes.

## Costing Subanalysis

The costing analysis followed an established micro-costing methodology [17, 18]. The total cost of care was calculated using electronic medical records for patients with falciparum malaria treated as outpatients. The cost of care and number of nights spent in the hospital were then calculated for a control group for comparison.

### Participants

The cost analysis considered patient data from a subsample of the overall analysis composed of all participants from GSTT and HTD from the study period, for whom detailed patient cost records were readily obtainable. One patient was excluded from this subanalysis because they were initially investigated for viral hemorrhagic fever, and their treatment costs primarily reflected VHF screening. This resulted in 61 participants from the overall analysis included in the outpatient group of the costing subanalysis.

### Analysis Methods

Micro-data on investigations and treatment provided to each patient were extracted from electronic medical records, then used to calculate the cost of each recorded aspect of the malaria care pathway for each patient. All costings are based on the latest available national data from the NIHR Interactive Costing Tool 2024–25 and the NHS National Schedule of Costs [19, 20]. The median cost of care and difference in sample populations were compared across the 2 groups through summary statistics.

Where medical record data did not directly map onto the national cost data sets, 2 clinicians determined the most appropriate equivalent item. This means cost estimates likely represent an upper bound, as some of these items were listed as less expensive in local records. However, the same national cost data set was applied throughout for external validity. Cost per item is included in Supplementary Table 2.

Clinic appointment costs were recorded as tropical medicine clinics [19]. If patients were scheduled for an appointment but did not attend, the full cost of this appointment was included. Where patients were also followed up for unrelated conditions, costs assessed by 2 clinicians as reasonably attributable to malaria were recorded. Equally, where patients were first assessed in a clinical setting outside the trust, only care costs applicable to the trust in our study were included.

Baseline statistics, expressed as proportions, were compared between the outpatient and control arms using LibreTexts statistics. A cost analysis was undertaken using Microsoft Excel, version 16.92, with median (interquartile range [IQR]) overall cost and cost subcategories compared between arms using the Mann-Whitney *U* test [21–23].

### Control Group Identification

A control group for the cost analysis consisted of nonsevere malaria cases admitted overnight for nonclinical reasons, who thus were otherwise similar to those treated as outpatients. The assessment that admission was only for nonclinical reasons was made by 2 clinicians in parallel (of A.W., G.O.H., J.M., and A.C.). Reasons included cases where presentation late in the day meant the malaria film was returned overnight or where guidelines were misinterpreted, resulting in unnecessary admission.

## RESULTS

**Figure 1.**
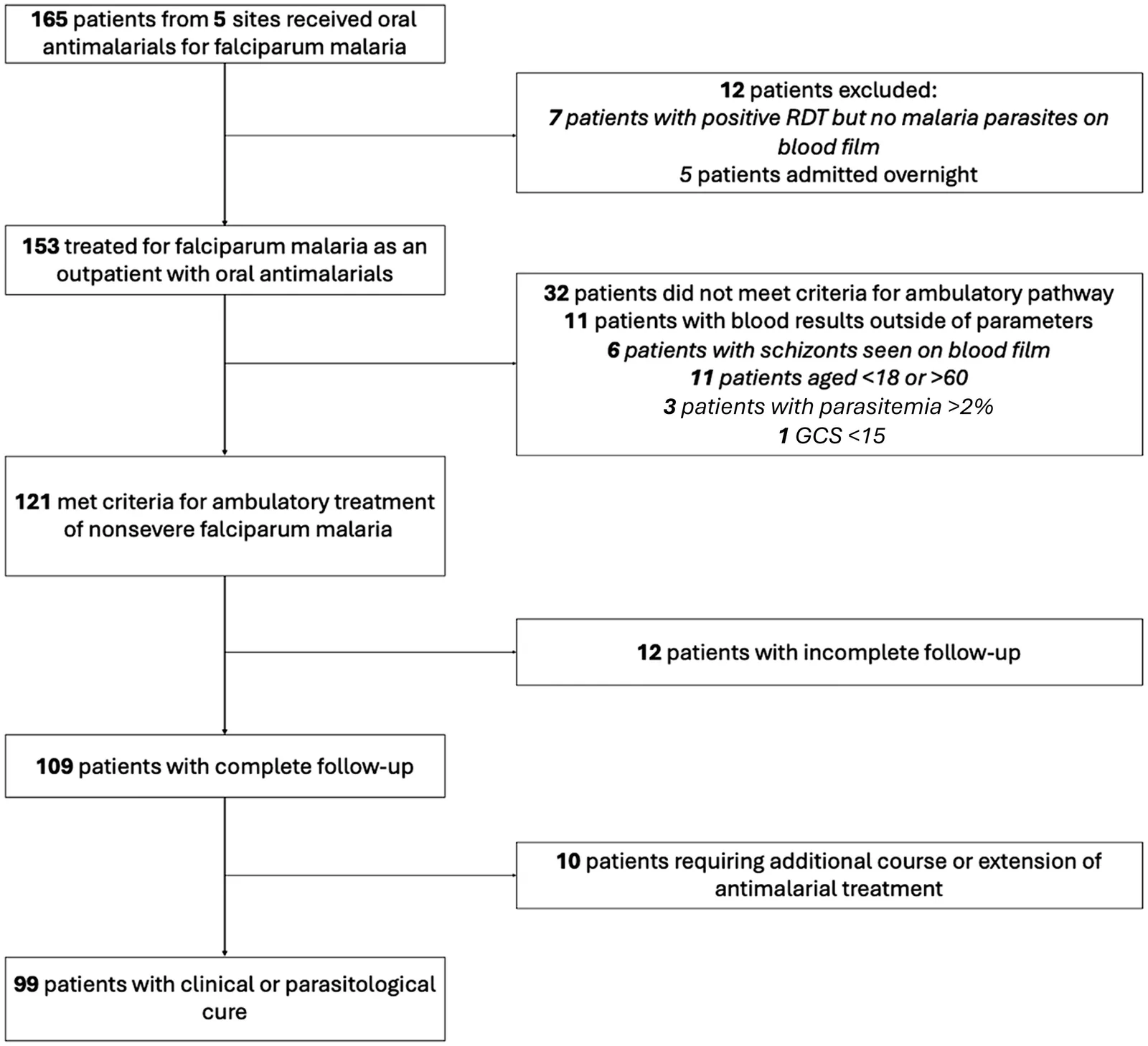
CONSORT diagram for patient inclusion and summary of patient outcomes. Abbreviations: GCS, Glasgow Coma Scale; RDT, rapid diagnostic test.

Between December 2019 and December 2024, 121 patients with confirmed *P. falciparum* infection who met guideline criteria were treated as outpatients with oral antimalarials (Figure 1). Baseline characteristics of included patients are detailed in Table 2. Region of travel was known in 96% (116/121) of participants, with West Africa being the most common region at 69% (80/116). Reason for travel was known in 90% (109/121) of patients, among whom 76% (92/109) were visiting friends and relatives.

**Table 2.** Baseline Clinical, Ethnic, and Laboratory Characteristics of Included Patients

Demographic Variable	Patients
	No. (%), Except Where Otherwise Stated
Age, median (IQR), y	48 (35–55)
Sex, % (No.)	
Female	44 (36)
Male	77 (64)
Comorbidities, % (No.)	
Chronic obstructive pulmonary disease	1 (1)
Diabetes – non-insulin-dependent	12 (10)
Diabetes – insulin-dependent	0
Heart failure	1 (1)
HIV (well controlled)	3 (2)
HIV (poorly controlled or CD4 <200)	0
Vomiting before treatment, % (No.)	
Yes	17 (14)
No	98 (81)
Not reported	6 (5)
Duration of symptoms, median (IQR), d	5 (3–7)
Region of birth, % (No.)	
Europe or North America	10 (8)
Central Africa	13 (11)
East Africa	15 (12)
West Africa	57 (47)
Unknown	26 (21)
Region of travel, % (No.)	
Central Africa	17 (14)
East Africa	17 (14)
North Africa and West Asia	2 (2)
West Africa	80 (66)
Unknown	5 (4)
Duration of travel, % (No.)	
≤7 d	3 (2)
8–21 d	34 (28)
>21 d	61 (50)
Unknown	19 (21)
Reason for travel	
Business	9 (7)
Relocating from malaria-endemic country to UK	1 (1)
Tourism	4 (3)
Visiting friends and relatives	92 (76)
Volunteer	1 (1)
Other	2 (2)
Unknown	12 (10)
Laboratory results	
Median hemoglobin (IQR), g/L	132 (121–142)
Median platelet count (IQR), 10^9^/L	124 (79–177)
Median creatinine (IQR), µmol/L	86 (73–105)
Median bilirubin (IQR), µmol/L	15 (9–23)
Median glucose (IQR), mmol/L	6.0 (5.3–6.9)
Median parasitemia (IQR), %	0.1 (0.1–0.6)

Abbreviation: IQR, interquartile range.

All included patients had a malaria blood film with *P. falciparum* observed on microscopy. The median parasitemia on presenting blood film (IQR) was 0.1% (0.1%–0.6%). Preschizonts were reported in 4% (5/121) patients, but all patients with schizonts were excluded. There were 3 cases of mixed infection; 2 cases of *P. ovale* and *P. falciparum* and 1 case of *P. malariae* and *P. falciparum*.

A total of 90% of patients (109/121) were followed up with a clinical review and/or repeat malaria film. All patients were prescribed a standardized, weight-based 3-day course of artemether-lumefantrine (AL). Adherence to treatment was recorded in 97% (106/109). Where adherence was recorded, 94% (100/106) reported missing no doses; 2% (2/106) reported missing 1 dose, 2% (2/106) reported missing 2 doses, and 2% (2/106) reported missing 3 or more doses. Vomiting during the treatment course was reported in 4% (4/106).

Clinical or parasitological cure within 2 weeks was observed in 90% (99/109) of patients who received 3 days of AL and were followed up with either a clinical review or repeat malaria film.

Among the 10 participants who had no evidence of clinical or parasitological cure following treatment, 4 patients received a repeat course of oral antimalarials outpatient, 4 patients were readmitted for treatment, and 2 patients were not retreated or readmitted. In the same group of patients without evidence of clinical or parasitological cure, 50% (5/10) were either unable to take the treatment or were not fully adherent to the prescribed treatment, and 5 patients were fully adherent and tolerated the full course of treatment.

Among those who were fully adherent to treatment and did not have evidence of clinical or parasitological cure: 2 patients had a repeat malaria film early in the treatment course with a persistent but improving parasitemia (they did not receive further treatment but were not reviewed by clinicians again), 1 participant had a recrudescence of malaria 3 weeks after treatment and was prescribed atovaquone-proguanil, 1 patient had a persistent parasitemia with ongoing symptoms despite treatment and was admitted for intravenous artesunate, and 1 patient had a persistent parasitemia and received a repeat course of AL. There were no admissions requiring high-dependency or intensive care, and there were no deaths.

There were 2 further patients who received additional treatment with AL despite evidence of clinical and parasitological cure. One participant vomited multiple times during the course of treatment, and a clinical decision was made to extend treatment. The other patient had a recurrence of symptoms 2 weeks after treatment with a negative blood film, and the treating clinicians chose to repeat treatment.

There were 32 patients who received ambulatory therapy despite meeting exclusion criteria for the main analysis, among whom 28 had follow-up, who were not included in the core analysis. None of the patients who were followed up were admitted to high-dependency or intensive care, and there was no mortality. There was evidence of clinical or parasitological cure in 85% (24/28), and 11% (3/28) required additional retreatment; 1 of these patients required admission for a course of intravenous (IV) artesunate.

### Cost Analysis

This analysis included all patients with nonsevere malaria treated on an outpatient pathway (“outpatient”; n = 61) at GSTT and HTD, the 2 trusts with full micro-costed electronic health records available. It also included all patients with nonsevere malaria admitted as inpatients for nonclinical reasons in the same 2 trusts over the study period (“overnight”; n = 11). The outpatient and overnight groups were comparable on all key demographic and clinical parameters at admission, aside from age, for which the median and IQR were slightly lower in the overnight control group (*P* = .038) (Table 3). One patient treated as an outpatient then returned and was subsequently admitted; this patient was still classified as being managed under the “outpatient pathway” following an intention-to-treat approach.

**Table 3.** Comparison of Baseline Characteristics Between Arms of Costing Subanalysis; *P* Values for Gender and Ethnicity Calculated Using LibreTexts χ^2^ Test for Independence [23]; Comparisons of Medians Calculated Using Mann-Whitney *U* Test in Microsoft Excel, Version 16.92

Cost Component	Outpatient (n = 61), No. (%)	Overnight (n = 11), No. (%)	*P* Value
Gender			
Male	41 (67.2)	9 (81.8)	.33
Female	20 (32.8)	2 (18.2)	
Ethnicity			
Black and minority ethnic	42 (68.9)	8 (72.7)	.73
White/Asian/mixed	8 (13.1)	2 (18.2)	
Ethnicity not stated	11 (18.0)	1 (9.1)	
Median age at diagnosis (IQR), y	49 (38–55)	38 (36–44)	.04
Laboratory results			
Median hemoglobin (IQR), g/L	132 (121–142)	138 (127–142)	.56
Median platelet count (IQR), 10^9^/L	139 (87–189)	116 (93–161)	.60
Median creatinine (IQR), µmol/L	86 (75–104)	98 (80–115)	.40
Median bilirubin (IQR), µmol/L^a^	16 (9–24)	24 (11–33)	.18
Median parasitemia (IQR), %	0.10 (0.004–0.30)	0.10 (0.02–0.35)	.78
Comorbidities			
Chronic obstructive pulmonary disease	0 (0)	0 (0)	1.00
Diabetes – non-insulin-dependent	5 (8.2)	0 (0)	.45
Diabetes – insulin-dependent	0 (0)	0 (0)	1.00
HIV (well controlled)	0 (0)	0 (0)	1.00
HIV (poorly controlled or CD4 <200)	0 (0)	0 (0)	1.00

Abbreviation: IQR, interquartile range.

^a^One bilirubin result in outpatient arm hemolyzed (GSTT ID 003).

The estimated median cost for the outpatient pathway was £1248, compared with a median cost for those admitted of £1792 (Table 4). The overnight group spent a median of 1 night in the hospital, compared with a median of 0 nights for the control group.

**Table 4.** Comparison of Costs Comparing Outpatient and Overnight “Control” Groups for Costing Subanalysis

Cost Component	Outpatient (n = 61), Median Cost (IQR), £	Overnight (n = 11), Median Cost (IQR), £	*P* Value
Overall cost	**1248** (**1039–1424)**	**1792** (**1593–2029)**	**<**.**001**
Investigations	681 (549–894)	910 (747–1125)	.014
Treatments	23 (23–23)	24 (23–32)	<.001
Admission (bed days)	0 (0–0)	510 (510–544)	<.001
Clerking	171 (153–334)	153 (153–162)	.305
Follow-up appointments (attended by patient)	227 (163–327)	0 (0–227)	.067
Follow-up appointments (patient did not attend)	0 (0–163)	0 (0–0)	.381
Nights in hospital	**0 (0–0)**	**1 (1–1)**	**<.001**

Abbreviation: IQR, interquartile range.

## DISCUSSION

Outpatient management of nonsevere falciparum malaria in the United Kingdom, a non-malaria-endemic country, has been successfully integrated into the clinical care pathways of 5 busy, urban hospitals, with 1 site having followed this model using a locally developed guideline since 2007. Initial outpatient management was safe and clinically effective and resulted in significant cost savings, due to reduced investigation costs and shorter length of hospital stay. It represents a practical and acceptable treatment for patients with nonsevere falciparum malaria in the United Kingdom. However, ambulatory pathways are reliant on robust outpatient follow-up as a small but important subset of patients required additional antimalarial treatment.

In this study, 121 patients with nonsevere falciparum malaria met criteria for outpatient management and were treated with a standard 3-day course of oral AL. Most patients were treated successfully and needed no further clinical input. Approximately 1 in 10 patients required an extension or repeat course of antimalarials, in most cases due to a clear and identifiable reason for treatment failure. Vomiting or nonadherence with prescribed treatment was responsible for treatment failure in half of those requiring additional treatment. Despite only a relatively small proportion of patients (4%) reporting vomiting on presentation, nausea and vomiting are commonly recognized side effects of oral antimalarials, and observation of patients after the first dose of treatment is essential to ensure that the drug is well tolerated. Among the 5 patients where no identifiable cause for treatment failure was observed, 2 of these patients had traveled to Uganda; the others had traveled to Sierra Leone and Democratic Republic of Congo. The emergence of partial artemisinin resistance is well documented in Uganda but was not recorded at the conception of the guidelines and may have contributed to AL failure [24, 25].

Although this study was not powered to detect mortality risk, there were no patients with an adverse outcome including admission to high-dependency or intensive care, and no deaths. Outpatient treatment of nonsevere falciparum malaria appears safer than other more common conditions that are routinely treated outpatient. Community-acquired pneumonia is routinely managed on an outpatient basis in patients stratified to a predicted mortality risk of up to 7.6% based on CURB scoring [26]. Furthermore, there were an additional 28 patients who received ambulatory therapy despite meeting exclusion criteria for outpatient management due to having clinical or laboratory parameters in keeping with more severe disease; these patients were followed up. The safety profile of this group was comparable with the core patient group meeting criteria for ambulatory treatment. Consensus guidelines developed for this study are relatively conservative, and there may be scope to treat more patients on an ambulatory pathway as outpatient management becomes more established in nonendemic countries.

This is the first study we are aware of that combines clinical outcomes with cost analysis of outpatient treatment for falciparum malaria. Treating nonsevere falciparum malaria through an outpatient pathway resulted in cost savings of £544/patient, a 30% reduction when compared with inpatient treatment of a comparable patient group, and saved a median of 1 night in the hospital for the patient. The control arm of patients admitted overnight for nonclinical reasons was small (n = 11), but the effect size was large, so cost savings were significant at the 95% level. The outpatient group also saw significantly lower costs for the subcategories of investigations and admissions (bed days).

The subcategory of “treatments” had an almost identical median cost and IQR; however, the Mann-Whitney test was statistically significant, reflecting a difference in the overall cost distribution driven by an extreme outlier in the outpatient arm. This is because of the single participant in the outpatient group who was initially managed as an outpatient then subsequently admitted for IV artesunate, which incurred substantially higher costs. Even with this outlier, the median treatment cost was virtually identical between groups.

The 2 patient groups were similar on all characteristics considered aside from age, as the “overnight” control group was slightly younger. This may have attenuated the estimated effect size, as younger admitted patients may be less likely to incur additional costs of care or complications such as severe hospital-acquired infections. Nonetheless, this analysis presents a strong economic case for careful, guideline-driven application of an outpatient malaria pathway. It also demonstrates the feasibility of a micro-costing approach and identification of natural controls to augment an NHS clinical outcomes study.

Although we did not collect specific employment or financial details of the patient cohorts, we note that inpatient management for health conditions can incur significant direct and indirect costs to both patients and their households. Length of stay for malaria-related hospitalizations is often used as a proxy for lost productivity and/or foregone earnings for patients [27, 28].

The average weekly earnings in the United Kingdom in August 2024, the last month for which study data were collected, were £697, implying average daily earnings of £139 for a 5-day work week [29]. Our finding of a median additional night in the hospital for the overnight “control” group suggests that inpatient care could increase indirect patient costs and inconvenience if the patients do not otherwise need to be in the hospital. However, this should be interpreted with caution as not all patients treated on an outpatient pathway would return to work immediately. Moreover, the outpatient group incurred higher median direct costs for follow-up appointments, which could also carry associated indirect costs and time burden.

There are a number of limitations to this study. Approximately 11% of patients were lost to follow-up, an expected proportion given that the population of individuals affected by malaria is highly mobile. We cannot comment on the outcome of this group. Missingness was assumed to be random and accounted for by performing complete case analysis. There was significant disruption to this study due to COVID, so the study population was smaller than anticipated due to reduced global travel. All laboratories at the participating sites are NEQAS accredited, but there may be differences in reporting between the sites; in particular, not all laboratories routinely report pre-schizonts, which may herald an increase in parasitemia and severe disease and therefore change how patient management is stratified. Similarly, turnaround times for reporting malaria films were not standardized across laboratories and were not systematically captured. While this does not directly impact the results of this study, it is an important consideration for the interpretation and implementation of these findings within different health care systems. Sufficient resources and time to allow standardization of laboratory processes and training in the clinical protocol would likely have improved the generalizability of these findings. In order to implement an outpatient treatment pathway, hospitals need to have a malaria film turnaround time in keeping with national guidance and the infrastructure to follow up patients in an outpatient setting [30]. All guidelines include some reference to presumed immunity. However, this was not reflected in clinical practice as 36% of patients receiving outpatient treatment either did not have the country of birth recorded or were not born in a malaria-endemic country. Therefore, patients born in nonendemic areas were included in this analysis, and further evaluation may be warranted to determine whether such patients could be considered for inclusion in outpatient treatment pathways within future guideline updates.

In conclusion, this study suggests that outpatient management of nonsevere falciparum malaria with robust outpatient follow-up offers a safe and effective alternative to routine hospital admission in appropriately selected patients. With updated evidence and growing clinical experience, aligning UK treatment pathways with widely established international guidance may reduce cost and ease pressure on health care services without compromising clinical safety.

## Supplementary Material

ofag443_Supplementary_Data

## Supplementary Data

Supplementary materials are available at *Open Forum Infectious Diseases* online. Consisting of data provided by the authors to benefit the reader, the posted materials are not copyedited and are the sole responsibility of the authors, so questions or comments should be addressed to the corresponding author.
