# Supplementary material for: Outpatient Management of Imported Nonsevere Falciparum Malaria in Adults in the United Kingdom: A Multisite Evaluation of Clinical Safety and Costing Analysis
Source: Open Forum Infect Dis. 2026 Jul 22;13(8):ofag443. doi: 10.1093/ofid/ofag443 (PMC13489155; doi:10.1093/ofid/ofag443)
Supplement: Supplementary file 1 [file ofag443_supplementary_data.docx]

**Supplementary materials**

|  | **_North Middlesex University Hospital_** | **_Guy’s and St Thomas’s NHS Foundation Trust_** |
| --- | --- | --- |
| ***_Age_*** | _>60_ | _<16 or ≥60_ |
| ***_Pregnancy_*** | _Pregnant people excluded_ | _Pregnant people excluded_ |
| ***_Immunity_***  *_Criteria variably described as inclusion or exclusion criteria rephrased as exclusion criteria_* | _Non-immune individuals_  *_Defined as not “born and brought up in malaria endemic region”_* | _Absence of semi-immunity_  _Defined as “continuous residence in malaria-endemic area for ≥15 years”_ |
| ***_Co-morbidities_*** | _Sickle cell disease/Splenectomy/Newly diagnosed or untreated HIV infection_ | _Sickle cell disease/Asplenia_ |
| ***_Laboratory parameters_*** | _Haemoglobin <80g/dL_  _Platelets <30 platelets/μL_  _Creatinine >120 μmol/L_  _Bilirubin >50 μmol/L_  _Glucose – no comment_ | _Haemoglobin <80g/dL_  _Platelets <30 platelets/μL_  _Creatinine >150 μmol/L_  _Bilirubin >50 μmol/L_  _Glucose no comment_ |
| ***_Parasitology results_*** | _>2% or schizonts_ | _≥2%_ |
| ***_Vomiting/Intolerance_*** | _Vomiting_ | _Vomiting and patients should be observed for 30 minutes after treatment_ |
| ***_Social circumstances_*** | _Family member or friend will be at home with patient_ | _No comment_ |

**Supplementary table 1:** Summary of guidelines for patients with non-severe falciparum malaria from two study sites with pre-existing guidelines; Guy’s and St. Thomas’ NHS Foundation Hospital and North Middlesex University Hospital

| **Item** | **Unit Cost** | **Source** | **Listing detail** |
| --- | --- | --- | --- |
| Hep A IgG Abs | £ 14.00 | NIHR 2025(1) |  |
| Hep A IgM | £ 14.00 | NIHR 2025 |  |
| Hep B surface Ag | £ 12.00 | NIHR 2025 |  |
| Hep B core Ab | £ 24.00 | NIHR 2025 |  |
| Hep B surface Ab | £ 18.00 | NIHR 2025 |  |
| Hep B core IgM | £ - |  | Included in above |
| Hep Be Ag | £ 20.00 | NIHR 2025 |  |
| Hep Be Ab | £ 20.00 | NIHR 2025 |  |
| Hep Be Ab interpretation | £ - |  | Included in above |
| Hep B viral load | £ 52.00 | NIHR 2025 |  |
| Hep C IgG Abs | £ 32.00 | NIHR 2025 | Hepatitis C antibody (HCVab) (anti-HCV) primary test |
| HIV ag/ab | £ 18.00 | NIHR 2025 |  |
| HTLV-1/HTLV-2 Abs | £ 64.00 | NIHR 2025 | HTLV1 (£32) + HTLV2 ( £32) |
| Filaria serology | £ 52.00 | NIHR 2025 | Infectious agent antigen detection by immunoassay technique' |
| Strongyloides serology | £ 52.00 | NIHR 2025 | Infectious agent antigen detection by immunoassay technique' |
| Schistosomal serology | £ 52.00 | NIHR 2025 | Infectious agent antigen detection by immunoassay technique' |
| Faeces for parasites | £ 20.00 | NIHR 2025 | Culture, pathogenic organisms, presumptive, screening only; including nasal swab' |
| Stool/faeces PCR | £ 20.00 | NIHR 2025 |  |
| Stool C difficile | £ 14.00 | NIHR 2025 |  |
| Infection serum - acute/convalescent/save | £ - |  | Additional cost applied when used for testing |
| Blood culture | £ 20.00 | NIHR 2025 |  |
| MRSA routine screen | £ 20.00 | NIHR 2025 | Culture, bacterial; throat or nose, aerobic with definitive identification |
| ED POCT CEPHEID COVID Flu | £ 20.00 | NIHR 2025 | Culture, bacterial; throat or nose, aerobic with definitive identification |
| Wound swab | £ 12.00 | NIHR 2025 | Culture, bacterial; any other source except urine, blood, serum, stool or feces, with isolation and presumptive identification of isolates |
| Throat swab - ENT | £ 20.00 | NIHR 2025 | Culture, bacterial; throat or nose, aerobic with definitive identification |
| Coagulation screen | £ 22.00 | NIHR 2025 | Any of 'Prothrombin time (PT)', 'Thrombin time; plasma (TT) (TCT) (Thrombin clotting time)', 'Thromboplastin time, partial (PTT) (aPTT); plasma or whole blood, serum' |
| Urine dip | £ 21.00 | NIHR 2025 | Urinalysis, non-automated |
| Urine culture | £ 12.00 |  |  |
| Midstream urine | £ 21.00 | NIHR 2025 | Urinalysis (urine analysis) (UA); bacteriuria screen, except by culture or dipstick |
| Urine pregnancy | £ 10.00 | NIHR 2025 |  |
| Urine protein | £ 18.00 | NIHR 2025 | 24h urine protein - spot not listed |
| Urine for schistosoma | £ 21.00 | NIHR 2025 | Urinalysis (urine analysis) (UA); microscopic only' |
| Blood film | £ 14.00 | NIHR 2025 | Blood count; manual differential WBC count (includes RBC morphology and platelet estimation) |
| Virus PCR (Ref Lab) | £ 52.00 | NIHR 2025 | Infectious agent detection by nucleic acid (DNA or RNA), not otherwise specified; amplified probe technique, each organism (e.g. PCR BK virus, PCR JC virus); urine, plasma, or serum; quantitative |
| Virus detection on EDTA blood | £ 52.00 | NIHR 2025 | Infectious agent detection by nucleic acid (DNA or RNA), not otherwise specified; amplified probe technique, each organism (e.g. PCR BK virus, PCR JC virus); urine, plasma, or serum; quantitative |
| FBC & Diff | £ 14.00 | NIHR 2025 | Blood count; hemogram and platelet count, automated and automated complete differential WBC count (CBC), haemogram, hematology, haematology ' |
| ESR | £ 8.00 | NIHR 2025 | Sedimentation rate, erythrocyte (ESR) (SED); non-automated |
| CRP | £ 14.00 | NIHR 2025 | CRP |
| Comprehensive metabolic panel | £ 99.00 | NIHR 2025 | Comprehensive metabolic panel: albumin, ALP, ALT (SGPT), AST (SGOT), bilirubin, blood urea nitrogen (BUN), calcium, carbon dioxide, chloride, creatinine, glucose, inorganic phosphate, potassium,sodium |
| Renal profile | £ 35.00 | NIHR 2025 | Recorded as 'Electrolyte panel: carbon dioxide, chloride, potassium, sodium' (26 pounds) + serum creatinine (9 pounds) |
| Liver profile | £ 26.00 | NIHR 2025 | LFT (Albumin, ALT, Total Bilirubin, Alk Phos) |
| Bone profile | £ 44.00 | NIHR 2025 | Calcium + albumin (12 pounds) + ALP (26, included in liver function tests) + phosphate (6 pounds) |
| TSH | £ 18.00 | NIHR 2025 | Thyroid stimulating hormone (TSH) |
| HbA1c level | £ 17.00 | NIHR 2025 |  |
| Glucose | £ 6.00 | NIHR 2025 | Glucose; blood, serum (except reagent strip); quantitative |
| Urea | £ 14.00 | NIHR 2025 | Urea nitrogen (BUN); blood, serum; quantitative |
| Creatinine | £ 9.00 | NIHR 2025 | Serum Creatinine |
| LDH | £ 9.00 | NIHR 2025 | LDH (Lactate Dehydrogenase) |
| Amylase | £ 10.00 | NIHR 2025 |  |
| D dimer | £ 8.00 | NIHR 2025 | Fibrin degradation products, D-dimer |
| Lactate | £ 9.00 | NIHR 2025 | Set as equal to other serum tests e.g. LDH, creatinine |
| Troponin T | £ 24.00 | NIHR 2025 | Troponin, quantitative; Cardiac Troponin I (cTnI), Cardiac Troponin T (cTnT) |
| Magnesium | £ 10.00 | NIHR 2025 |  |
| Bicarbonate | £ 14.00 | NIHR 2025 |  |
| Haptoglobin | £ 17.00 | NIHR 2025 |  |
| Reticulocytes | £ 14.00 | NIHR 2025 |  |
| CMV IgM | £ 98.00 | NIHR 2025 | CMV IGM (if reflexed) follow up |
| CMV IgG Abs | £ 98.00 | NIHR 2025 | CMV IGG |
| Anti-strep Abs | £ 52.00 | NIHR 2025 | Infectious agent detection by immunoassay with direct optical observation; Streptococcus, group A |
| Syphilis Abs | £ 32.00 | NIHR 2025 |  |
| Borrelia burgdorferi Abs | £ 32.00 | NIHR 2025 | Antibody; borrelia burgdorferi (lyme disease) |
| Alphavirus | £ 70.00 | NIHR 2025 | Chikungunya = IgA, IgG, IgM (18 pounds) + RNA (52 pounds; Infectious agent detection by nucleic acid (DNA or RNA), not otherwise specified; amplified probe technique, each organism (e.g. PCR BK virus, PCR JC virus); urine, plasma, or serum; quantitative) |
| Flavivirus | £ 72.00 | NIHR 2025 | IgG and IgM (18 pounds x4) for: dengue, West Nile, Tickborne encephalitis, Yellow fever |
| Leptospira | £ 52.00 | NIHR 2025 | Leptospira serology + IgM: Infectious agent antigen detection by immunoassay technique, (eg, enzyme immunoassay [EIA], enzyme-linked immunosorbent assay [ELISA], immunochemiluminometric assay [IMCA]) 52 pounds |
| Phlebovirus | £ 90.00 | NIHR 2025 | 5x IgG (18 pounds x5) |
| Rickettsia | £ 106.00 | NIHR 2025 | IgG/IgM 18x3, plus Rickettsial DNA (52 pounds; Infectious agent detection by nucleic acid (DNA or RNA), not otherwise specified; amplified probe technique, each organism (e.g. PCR BK virus, PCR JC virus); urine, plasma, or serum; quantitative) |
| Malaria parasites initial test | £ 252.00 | NIHR 2025 | Smear, primary source with interpretation; special stain bodies or intracellular parasites (eg, malaria, coccidia microsporidia, cytomegalovirus, herpes viruses) |
| Malaria parasites confirmation | £ - |  | Included in above |
| Malaria species PCR | £ 52.00 | NIHR 2025 | Infectious agent detection by nucleic acid (DNA or RNA), not otherwise specified; amplified probe technique, each organism (e.g. PCR BK virus, PCR JC virus); urine, plasma, or serum; quantitative |
| Iron & TIBC | £ 18.00 | NIHR 2025 | Iron (10) + TIBC (8) |
| Iron & transferrin | £ 26.00 | NIHR 2025 | Comprehensive Anemia Profile, Anaemia Panel including: reticulocyt count, Iron with TIBC, Vitamin B12, CBC with differential, ferritin, folate, folic acid. |
| B12 | £ 26.00 | NIHR 2025 | B12 and folate each' |
| Folate | £ 26.00 | NIHR 2025 | B12 and folate each' |
| Reticulocyte | £ 14.00 | NIHR 2025 |  |
| G6PD | £ 61.00 | NIHR 2025 | G6PD screen |
| Sickle cell and thalassaemia | £ 425.00 | NIHR 2025 | HBB (hemoglobin, beta) (eg, sickle cell anemia, hemoglobin C, hemoglobin E), common variants (eg, HbS, HbC, HbE) |
| Group and screen | £ 32.00 | NIHR 2025 | Antibody screen, RBC, antibody to specific drug, each serum technique |
| COVID asymptomatic | £ 14.00 | NIHR 2025 | SARS-COV-2 Quant IgG Antibody |
| COVID symptomatic | £ 14.00 | NIHR 2025 | SARS-COV-2 Quant IgG Antibody |
| Venous blood gas | £ 4.15 | NICE 2015(2) | The published 2015 price for a venous blood gas was £3 (2). This was inflated to £4.15 in 2025 prices using the UK Consumer Price Index (6) |
| bHCG | £ 14.00 | NIHR 2025 | Serum pregnancy, gonadotropin chorionic (hCG) (BetahCG); quantitative |
| Respiratory panel | £ 52.00 | NIHR 2025 | Infectious agent detection by immunoassay with direct optical observation; not otherwise specified |
| Chest X-ray | £ 36.79 | NHS 2025 | Directly Accessed Diagnostic Services - Plain Film. General Internal Medicine Service |
| Abdominal X-ray | £ 36.79 | NHS 2025 | Directly Accessed Diagnostic Services - Plain Film. General Internal Medicine Service |
| Staff cost taking blood | £ - |  | Included in item cost, per NIHR 2025 |
| Lateral flow COVID | £ 1.36 | NHSBSA 2023(3) | 6.80 for five tests |
| Lateral flow flu | £ 20.00 | NIHR 2025 | Culture, pathogenic organisms, presumptive, screening only; including nasal swab |
|  | | | |
| **Treatments** |  |  |  |
| Artemether 20mg + lumefantrine 120mg (24 tablet course) | £ 22.50 | BNF 2025(4) | Medicinal forms \| Artemether with lumefantrine \| Drugs \| BNF \| NICE |
| Atovaquone 62.5 mg, Proguanil hydrochloride 25 mg (12 tablet course) | £ 6.26 | BNF 2025 | https://bnf.nice.org.uk/drugs/atovaquone-with-proguanil-hydrochloride/medicinal-forms/#oral-tablet |
| 1L IV NaCl 0.9% | £ 1.89 | BNF 2025 | Sodium chloride 0.9% infusion 1litre Freeflex bags Fresenius Kabi Ltd https://bnf.nice.org.uk/drugs/sodium-chloride/medicinal-forms/#infusion |
| Paracetamol 1g | £ 0.05 | BNF 2025 | Medicinal forms \| Paracetamol \| Drugs \| BNF \| NICE |
| Omeprazole 20mg | £ 0.63 | BNF 2025 | Medicinal forms \| Omeprazole \| Drugs \| BNF \| NICE |
| Hartmann's | £ 3.64 | BNF 2025 | https://bnf.nice.org.uk/drugs/potassium-chloride-with-calcium-chloride-sodium-chloride-and-sodium-lactate/medicinal-forms/ |
| Cyclizine 1 tablet | £ 0.04 | BNF 2025 | https://bnf.nice.org.uk/drugs/cyclizine/medicinal-forms/ |
| Ibuprofen 400mg | £ 0.07 | BNF 2025 | https://bnf.nice.org.uk/drugs/ibuprofen/medicinal-forms/#oral-tablet |
| IV cefuroxime 1.5g | £ 4.70 | BNF 2025 | https://bnf.nice.org.uk/drugs/cefuroxime/medicinal-forms/ |
| IV artesunate 60mg | £ 82.67 | BNF 2025 | The published 2012 price for a five-vial course of IV artesunate was £287(5). This equates to a per-vial 2012 cost of £57.40, inflated to £82.67 in 2025 prices using the UK Consumer Price Index(6). |
| Paracetamol 1g IV | £ 1.25 | BNF 2025 | https://bnf.nice.org.uk/drugs/paracetamol/medicinal-forms/#solution-for-infusion |
| Codeine 30mg | £ 0.07 | BNF 2025 | https://bnf.nice.org.uk/drugs/codeine-phosphate/medicinal-forms/ |
| rivaroxaban 15mg | £ 1.81 | BNF 2025 | Indicative price 16.87 for 56 tablets |
| sacubitril/valsartan 49/51mg | £ 1.64 | BNF 2025 | £45.78 for 28 tablets |
| spironolactone 12.5mg | £ 0.43 | BNF 2025 | £12.00 for 28 |
| nebivolol 5mg | £ 0.15 | BNF 2025 | 4.30 for 28 tablets |
| furosemide 40mg | £ 0.02 | BNF 2025 | 0.59 for 28 tablets |
| cyclizine 50mg | £ 0.04 | BNF 2025 | 3.95 for 100 tablets |
| Seretide accuhaler | £ 17.46 | BNF 2025 | Per inhaler |
| brimonidine tartrate 0.2% | £ 2.54 | BNF 2025 | Per unit |
|  | | | |
| **Bed days** |  |  |  |
| Same-day emergency care | £ 171.01 | NHS 2025(7) | Consultant led mono specialty accident and emergency service (e.g. Ophthalmology, dental) with designated accommodation for the reception of patients', 'Any investigation with no significant treatment' |
| A&E presentation | £ 152.64 | NHS 2025 | Emergency departments are a CONSULTANT led 24 hour service, Emergency Medicine, No Investigation with No Significant Treatment |
| Non-elective inpatient - short stay | £ 543.67 | NHS 2025 | Non-elective inpatient - short stay: fever of Unknown Origin without Interventions, with CC Score 0-3 |
|  | | | |
| **Clinics** |  |  |  |
| Tropical Medicine Non-Admitted Face-to-Face Attendance, Follow-up | £ 226.51 | NHS 2025 |  |
| Tropical Medicine Non-Admitted Face-to-Face Attendance, First | £ 333.72 | NHS 2025 |  |
| Tropical Medicine Non-Admitted Non-Face-to-Face Attendance, Follow-up | £ 163.34 | NHS 2025 |  |
| Tropical Medicine Non-Admitted Non-Face-to-Face Attendance, First | £ 199.20 | NHS 2025 |  |

**Supplementary Table 2:** cost per item used for cost-benefit analysis

| **Patient ID** | **Clinical details** |
| --- | --- |
| *GSTT 1* | Given PO artemether-lumefantrine, admitted overnight |
| *GSTT 2* | Stayed overnight on ward due to late night presentation |
| *GSTT 3* | Admitted due to misapplied guidelines. Notes state CRP rising and platelets 43, neither exceeded guideline threshold for admission. |
| *GSTT 4* | Stayed overnight on ward due to late night presentation |
| *UCLH 1* | ‘No features of severity’, given PO artemether-lumefantrine but admitted for one night |
| *UCLH 2* | Parasites 0.15%, 'late trophozoites' on film (not a criteria for admission). Given PO artemether-lumefantrine, admitted overnight |
| *UCLH 3* | Clinic notes state 'not suitable for outpatient management at this point as renal function/ platelets etc not back yet' then transferred to ED and admitted overnight |
| *UCLH 4* | Parasites 0.2%, admitted ‘because pH 7.305’ (guidelines to admit if <7.3) |
| *UCLH 5* | 0.08% parasites, 'admitted for observation given late trophozoites' (not a criterion for admission) |
| *UCLH 6* | 0.2% parasites, systemically well, on artemether-lumefantrine. Patient 'not keen to go home just yet, feels fever is coming back'. Discharged next day. |
| *UCLH 7* | 0.01% parasites + late trophozoites, started artemether-lumefantrine, discharged next day |

***Supplementary table 3:*** *Non-clinical reasons for admission as assessed by two members of study team*

Additional notes on inclusion and costing judgments:

- One patient included this study was initially assessed and diagnosed at a different hospital emergency department. As the receiving centre repeated diagnostic investigations and initiated treatment independently, costs from the initial assessment at the referring hospital were not included in this analysis.
- One patient was initially managed as an outpatient but subsequently presented to their general practitioner with vomiting and then was prescribed atovaquone-proguanil in the community. As this treatment was initiated and costed within primary care, it fell outside the scope of this hospital-based costing analysis and could not be captured within the same costing framework. This patient was therefore excluded from the analysis.

**References**

1.	NIHR Interactive Costing Tool. interactive-costing-tool-tariff-data-2025-26-v1-test. Accessed February 2025.

2.	National Clinical Guidelines Centre. Preoperative tests. https://www.nice.org.uk/guidance/ng45/documents/guideline-appendices-13. Accessed February 2025.

3.	NHSBA Drug Tariffs November 2023. https://www.nhsbsa.nhs.uk/sites/default/files/2023-10/Drug%20Tariff%20November%202023.pdf. Accessed February 2025.

4.	BNF Drug Monographs. https://bnf.nice.org.uk/drugs/. Accessed February 2025.

5.	Broderick C, Friend P, Smith V, et al. Geographical concentration of falciparum malaria treated in the UK and delay to treatment with artesunate in severe cases: an observational study. BMJ Open 2012;2:e001854. <https://bmjopen.bmj.com/content/2/6/e001854>

6.	Office for National Statistics, Series D7BT: CPI INDEX 00: ALL ITEMS 2015=100. Release date 22 April 2026. <https://www.ons.gov.uk/economy/inflationandpriceindices/timeseries/d7bt/mm23>. Accessed 9 May 2026.

7.	National Reference Costs 2024/25. https://www.england.nhs.uk/costing-in-the-nhs/national-cost-collection/#:~:text=The%20National%20Cost%20Collection%20publication,the%20cost%20of%20NHS%20services. Accessed February 2025.
